# α-Lipoic acid suppresses p53 by preventing annexin A2 degradation to protect dopaminergic neurons in a Parkinson's disease model

**DOI:** 10.3389/fnins.2026.1837965

**Published:** 2026-05-08

**Authors:** Ying Liu, WenPing Sun

**Affiliations:** Department of General Practice, Songjiang Hospital Affiliated Shanghai Jiao Tong University School of Medicine, Shanghai, China

**Keywords:** annexin A2, degeneration, p53, Parkinson's disease, protection, α-lipoic acid

## Abstract

**Objective:**

p53 plays a critical role in Parkinson's disease (PD) pathogenesis. p53 activation induces mitochondrial dysfunction and reactive oxygen species (ROS) production, contributing to progressive dopaminergic neuron degeneration. Although α-lipoic acid (ALA) exhibits neuroprotective effects in neurodegeneration, its underlying mechanisms remain unclear. This study investigated the neuroprotective role and molecular mechanism of ALA in 1-methyl-4-phenylpyridinium (MPP^+^)- treated PC12 cells, a cellular model of dopaminergic toxicity.

**Methods:**

Neuroprotective effects of ALA on dopaminergic neurons were assessed using the 3-(4,5-dimethylthiazol-2-yl)-2,5-diphenyltetrazolium bromide (MTT) assay for cell viability, Hoechst 33258 staining and flow cytometry to detect cellular apoptosis, and western blot analysis.

**Results:**

ALA treatment significantly inhibited p53 expression and attenuated MPP^+^-induced apoptosis in dopaminergic neurons. ALA also prevented annexin A2 degradation and protected PC12 cells from MPP^+^-induced toxicity.

**Conclusions:**

ALA downregulates p53 expression by preventing annexin A2 degradation, thereby reducing p53 protein levels and providing neuroprotection under neurodegenerative conditions. This suggests the potential of ALA in modulating p53 pathways for PD therapy.

## Introduction

Parkinson's disease (PD) is a common neurodegenerative disorder characterized by the progressive degeneration of dopaminergic neurons in the substantia nigra pars compacta (SNpc) of the midbrain (Raza et al., 2019). Current treatments primarily restore dopamine levels to temporarily alleviate motor symptoms but fail to stop or delay the underlying progressive neurodegeneration. Mitochondrial dysfunction and oxidative stress are central events in PD pathogenesis (Liu et al., 2025; Blagov et al., 2024).

Mitochondria, multifunctional subcellular organelles, are primary cellular sources of reactive oxygen species (ROS). Mitochondrial dysfunction generates excessive ROS, causing oxidative damage to mitochondria; this further impairs function, leading to more ROS generation and creating a vicious cycle of uncontrolled oxidative stress associated with dopaminergic neuron degeneration in PD (Guo et al., 2018). The interplay between mitochondrial dysfunction and oxidative stress involves numerous regulatory proteins and signaling pathways, with p53 playing a critical role (Luo et al., 2022). Through transcription-dependent and -independent mechanisms, p53 overexpression and overactivation profoundly impact dopaminergic neuron degeneration by mediating mitochondrial dysfunction and increasing ROS production (Kim et al., 2024). p53 mitochondrial translocation triggers mitochondrial permeability transition pore (mPTP) formation, Ca^2+^ overload, aberrant α-synuclein accumulation, mitophagy impairment, and ROS production, collectively leading to mitochondrial dysfunction, oxidative damage, and ultimately cell death (Luo et al., 2022; Wang et al., 2024). Additionally, nuclear p53 translocation induces oxidative stress by activating specific target genes involved in ROS generation (Liu et al., 2020). Consequently, repressing p53 overexpression and overactivation represents a potential therapeutic strategy to protect dopaminergic neurons from mitochondrial dysfunction and oxidative stress-mediated cell death.

α-Lipoic acid (ALA), a well-known antioxidant, has recognized neuroprotective effects on dopaminergic neurons in neurodegenerative contexts (Li et al., 2016). This study investigated the neuroprotective effects of ALA and underlying mechanisms against MPP^+^-induced toxicity in cellular models of PD. Our results demonstrate that ALA effectively protected dopaminergic cells from 1-methyl-4-phenylpyridinium (MPP^+^) toxicity by inhibiting p53 overactivation, thereby attenuating mitochondrial dysfunction and associated neurodegeneration. Furthermore, ALA treatment prevented the degradation of the antioxidant protein annexin A2. As annexin A2 is known to inhibit p53 expression and activity (Wang et al., 2012; Wu et al., 2019), its stabilization by ALA leads to p53 inhibition, thereby reducing ROS production and attenuating neuronal death. These findings indicate that the neuroprotective effects of ALA are mediated by stabilization of annexin A2 and subsequent inhibition of p53 activation, thus identifying a novel mechanistic pathway and highlighting the potential of ALA as a therapeutic strategy for PD.

## Materials and methods

### Drugs and chemicals

All reagents and chemicals were purchased from Sigma-Aldrich (St. Louis, MO, USA) unless otherwise specified.

### Cell cultures

The rat adrenal pheochromocytoma cell line PC12 was purchased from the Cell Bank of the Chinese Academy of Sciences (Shanghai, China). Cells were cultured in high-glucose Dulbecco's modified Eagle's medium (DMEM) supplemented with 10% inactivated fetal bovine serum (Gibco, Grand Island, NY, USA), 100 U/ml penicillin, and 100 μg/ml streptomycin. Cultures were maintained at 37 °C in a humidified 5% CO_2_ incubator.

### Cell viability assay

Cell viability was assessed using the 3-(4,5-dimethylthiazol-2-yl)-2,5-diphenyltetrazolium bromide (MTT) assay. MTT is a cell-permeable, positively charged tetrazolium dye reduced by mitochondrial dehydrogenases to form insoluble purple formazan crystals in metabolically active cells. PC12 cells were plated at a density of 30,000 cells/cm^2^ in 96-well plates and incubated in a humidified 5% CO_2_ atmosphere at 37 °C. To evaluate the protective effects of ALA against 1-methyl-4-phenylpyridinium (MPP^+^), cells were pretreated with various concentrations of ALA (0.01–100 μM) for 3 h, followed by exposure to 2 mM MPP^+^ for 48 h; these conditions were optimized as previously described (Li et al., 2021). After treatment, MTT solution (5 mg/mL) was added to each well, and cells were incubated for 3 h at 37 °C. The medium was carefully aspirated, and formazan crystals were dissolved in dimethyl sulfoxide (DMSO). Absorbance was measured at 570 nm using a microplate reader (Epoch 2, BioTek, Winooski, VT, USA). The results were expressed as a percentage of the control group. The experiment was repeated three times.

### Nuclear staining assay

Apoptosis in MPP^+^-treated PC12 cells was evaluated using Hoechst 33258 staining. Following treatment, cells were washed and resuspended in phosphate-buffered saline (PBS), then stained with 10 μg/mL Hoechst 33258 for 10 min at room temperature in the dark. Nuclear morphology was examined under a fluorescence microscope (IX71; Olympus Corp., Tokyo, Japan). Apoptotic cells were identified by chromatin condensation, nuclear fragmentation, and pyknotic nuclei. The percentage of apoptotic cells was calculated relative to the total number of cells. Experiments were performed in triplicate.

### Flow cytometric analysis of apoptosis

Apoptosis of PC12 cells was analyzed by flow cytometry with Annexin V-FITC/propidium iodide (PI) double staining. PC12 cells were treated with the indicated concentrations of MPP^+^, while untreated cells served as the control group. After incubation, cells were harvested, washed twice with PBS, and centrifuged at 300 × g for 5 min at 4 °C. Cells were resuspended in binding buffer, followed by the addition of 5 μL Annexin V-FITC (20 μg/mL) and 5 μL PI (50 μg/mL). After incubation in the dark for 15 min at 4 °C, the samples were then analyzed immediately by flow cytometry (Becton-Dickinson, San Diego, CA, USA). Living cells (Annexin V^−^/PI^−^, Q3), early apoptotic cells (Annexin V^+^/PI^−^, Q4), late apoptotic cells (Annexin V^+^/PI^+^, Q2), and necrotic cells (Annexin V^−^/PI^+^, Q1) were distinguished. Therefore, the total apoptotic proportion included the percentage of Annexin V^+^/PI^−^ and Annexin V^+^/PI^+^ cells.

### Western blot analysis

Cultured cells were harvested and lysed using a cell lysis solution containing 50 mM Tris-HCl (pH 6.8), 4% sodium dodecyl sulfate (SDS), and 2 mM EDTA. The cell lysates were centrifuged and the protein concentrations were determined using a bicinchoninic acid (BCA) protein assay kit (Thermo Fisher Scientific, Waltham, MA, USA). Equal amounts of protein were loaded onto 12% SDS-polyacrylamide gels, separated by SDS-PAGE, and electrophoretically transferred onto polyvinylidene difluoride (PVDF) membranes. The membranes were blocked with 5% non-fat milk in Tris-buffered saline containing 1% Tween-20. Subsequently, the membranes were incubated overnight at 4 °C with the following primary antibodies diluted in blocking buffer: anti-p53 mouse monoclonal antibody (Cat. no. 554166; BD Biosciences) and anti-annexin A2 mouse monoclonal antibody (Cat. no. 610068; BD Biosciences). After washing three times with PBS, the membranes were incubated with horseradish peroxidase (HRP)-conjugated anti-mouse secondary antibody (Cat. no. R-21455; Pierce Biotechnology, Inc., Rockford, IL, USA). β-actin was used as a loading control. Protein bands were visualized using an enhanced chemiluminescence western blot detection kit (Engreen Biosystem, China) according to the manufacturer's instructions. For densitometric analysis, blot images were captured using a chemiluminescence imaging system (Bio-Rad, Hercules, CA, USA). Band intensities were quantified with ImageJ software (NIH, Bethesda, MD, USA). Each target protein band intensity was normalized to the corresponding β-actin band from the same lane, and the results were expressed as fold change relative to the control group. All experiments were repeated three times independently.

### Statistical analysis

Data are expressed as means ± SEM. Statistical analysis was performed using one-way analysis of variance, followed by Dunnett's multiple comparison test, using SPSS version 22.0 (IBM Corporation, Armonk, NY, USA). A value of *P* < 0.05 was considered statistically significant.

## Results

### ALA attenuates MPP^+^-induced loss of cell viability

**Figure 1:**
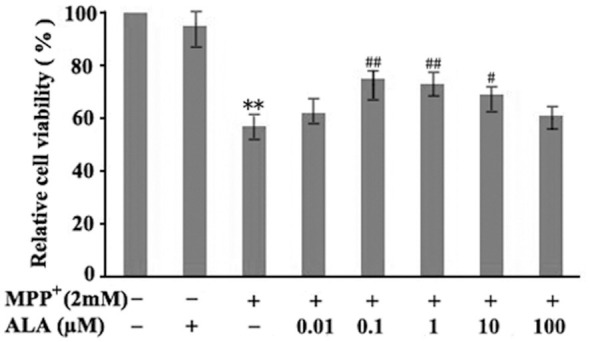
α-Lipoic acid (ALA) attenuates 1-methyl-4-phenylpyridinium (MPP^+^)-induced cytotoxicity in PC12 cells. PC12 cells were pretreated with ALA (0.01–100 μM) for 3 h, and then exposed to 2 mM MPP^+^ for 48 h. Cell viability was determined by MTT assay. Data are presented as the mean ± SEM of three independent experiments. **P < 0.01 vs. control; ^#^*P* < 0.05 or ^##^*P* < 0.01 vs. MPP^+^-treated group.

The protective effect of ALA against MPP^+^-induced cytotoxicity in PC12 cells was assessed by MTT assay. The results showed that exposure to 2 mM MPP^+^ for 48 h reduced cell viability to approximately 55% of the control level (45% decrease), representing a moderate toxicity level. This degree of cytotoxicity is optimal for detecting protective effects and avoids the irreversible damage caused by excessively high concentrations. Pretreatment with 0.1 μM ALA effectively attenuated the loss of cell viability induced by 2 mM MPP^+^ for 48 h, while treatment with ALA (0.1 μM) alone did not significantly alter cell viability (Figure 1). These results indicate that the cytotoxicity in PC12 cells induced by MPP^+^ was attenuated by ALA pretreatment.

### ALA exerts an anti-apoptotic effect against MPP^+^ toxicity

**Figure 2:**
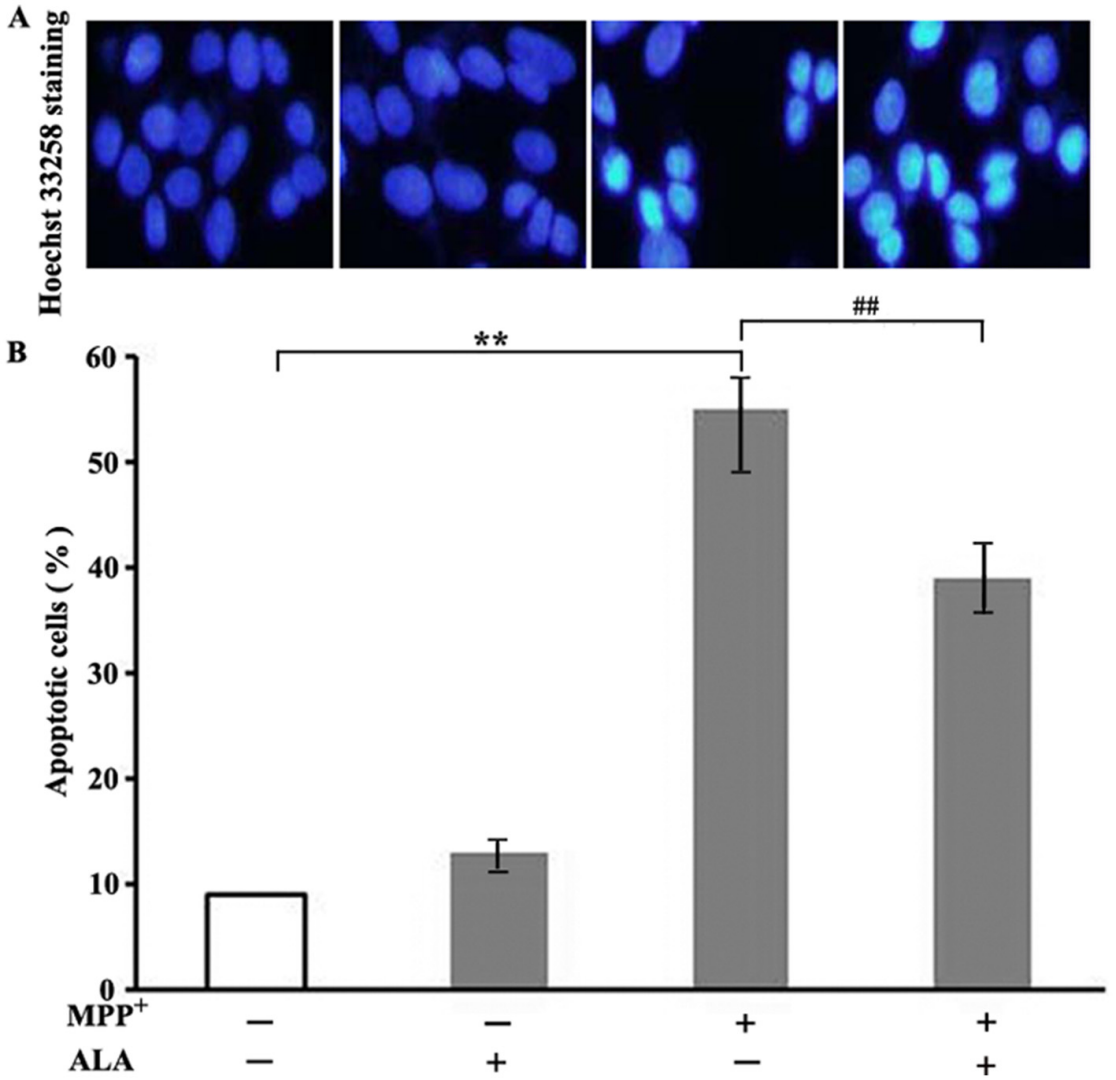
α-Lipoic acid (ALA) exerts an anti-apoptotic effect against MPP^+^ toxicity. PC12 cells were pretreated with 0.1 μM ALA for 3 h, and then exposed to 2 mM MPP^+^ for 48 h. **(A)** Apoptosis was quantified by Hoechst 33258 nuclear staining. **(B)** The histograms show the percentage of apoptotic cells. Data are presented as the mean ± SEM of three independent experiments. **P < 0.01 vs. control group; ^##^*P* < 0.01 vs. MPP^+^-treated group.

Apoptosis is characterized by a series of nuclear morphological changes that can be detected by using Hoechst 33258, a compound with high affinity for nucleic acids. Exposure to 2 mM MPP^+^ for 48 h markedly increased the percentage of apoptotic cells to 56% compared to that in the control group. In contrast, pretreatment with 0.1 μM ALA significantly reduced MPP^+^-induced apoptosis to 39%. Treatment with ALA alone (0.1 μM) did not significantly alter the apoptotic rate, supporting the protective role of ALA in MPP^+^-treated PC12 cells (Figure 2).

### ALA suppresses MPP^+^-induced apoptotic cell death

**Figure 3:**
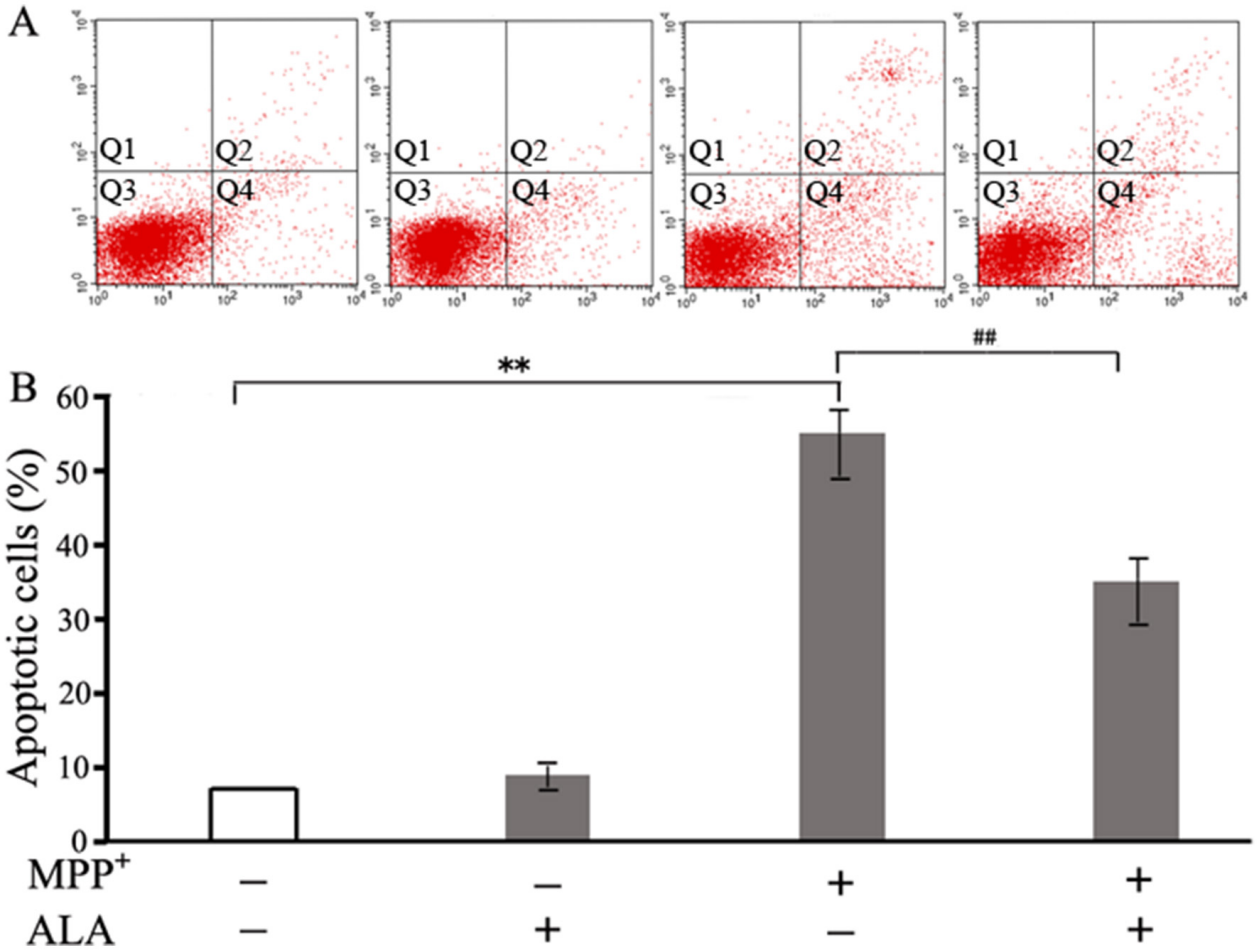
α-Lipoic acid (ALA) suppresses MPP^+^-induced apoptotic cell death. PC12 cells were pretreated with 0.1 μM ALA for 3 h, followed by exposure to 2 mM MPP^+^ for 48 h. **(A)** Apoptosis was analyzed by flow cytometry with Annexin V-FITC/propidium iodide (PI) double staining. **(B)** The histograms show the percentage of apoptotic cells. Data are presented as the mean ± SEM of three independent experiments. **P < 0.01 vs. control group; ^##^*P* < 0.01 vs. MPP^+^-treated group.

To determine whether ALA protects PC12 cells from MPP^+^-induced apoptosis, Annexin V-FITC/PI double staining was performed. Cells positive for Annexin V but negative for PI were defined as early apoptotic, whereas double-positive cells were considered late apoptotic. ALA alone did not significantly alter the number of apoptotic cells compared with the control group. In contrast, MPP^+^ markedly increased the apoptotic cell population to 57%, and this increase was significantly attenuated to 35% when ALA was administered 3 h prior to MPP^+^ exposure (Figure 3). These results confirmed the anti-apoptotic activity of ALA against MPP^+^-induced neurotoxicity.

### ALA represses p53 expression in MPP^+^-treated PC12 cells

**Figure 4:**
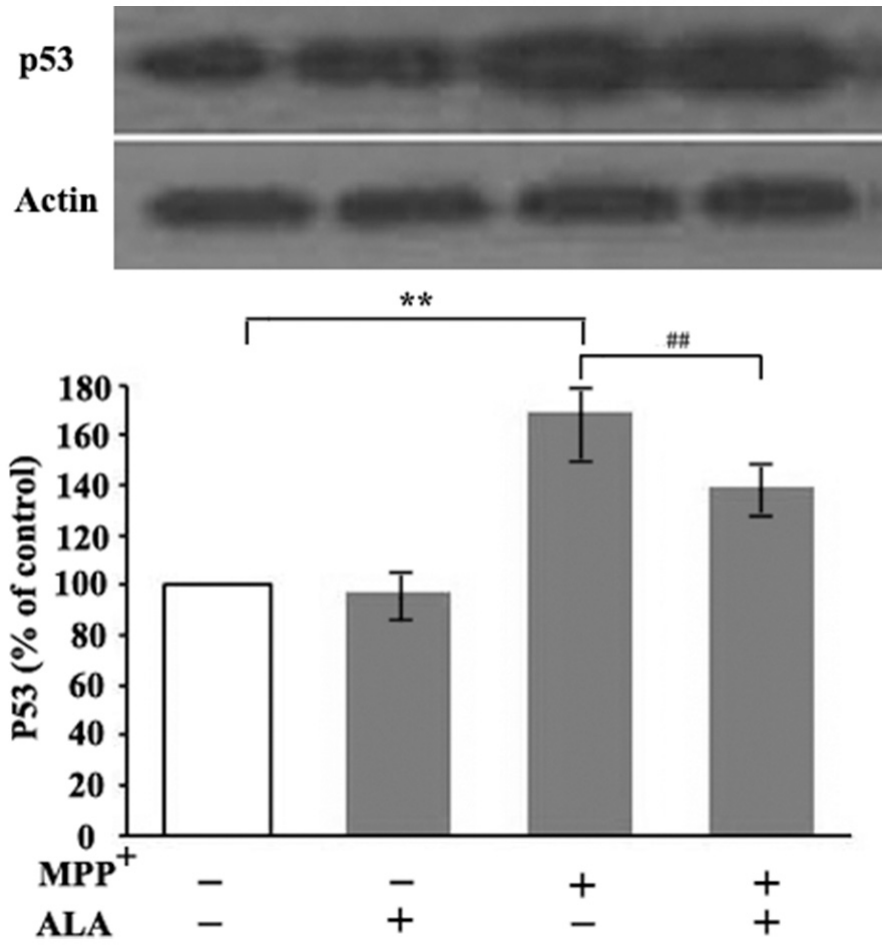
α-Lipoic acid (ALA) reduces p53 expression in 1-methyl-4-phenylpyridinium (MPP^+^)-treated PC12 cells. PC12 cells were either untreated or treated with 2 mM MPP^+^ alone or 2 mM MPP^+^ in the presence of 0.1 μM ALA. p53 protein levels were analyzed by western blot with β-actin as loading control. Data are presented as the mean ± SEM of three independent experiments. **P < 0.01 vs. control group; ^##^*P* < 0.01 vs. MPP^+^-treated group.

As p53 overexpression and overactivation are implicated in dopaminergic neuron degeneration in PD, we investigated whether ALA regulates p53 expression in a PD cell model. Treatment with 2 mM MPP^+^ for 48 h significantly increased p53 protein levels in PC12 cells, supporting p53 involvement in neurotoxicity under neurodegenerative conditions. Pretreatment with 0.1 μM ALA markedly reduced MPP^+^-induced p53 upregulation, while ALA alone did not alter basal p53 levels in the PD model cells. These data suggest that the protective effects of ALA on PC12 cells are at least partially mediated by the repression of p53 expression (Figure 4).

### Degradation of annexin A2 elevates p53 expression in MPP^+^-treated PC12 cells

**Figure 5:**
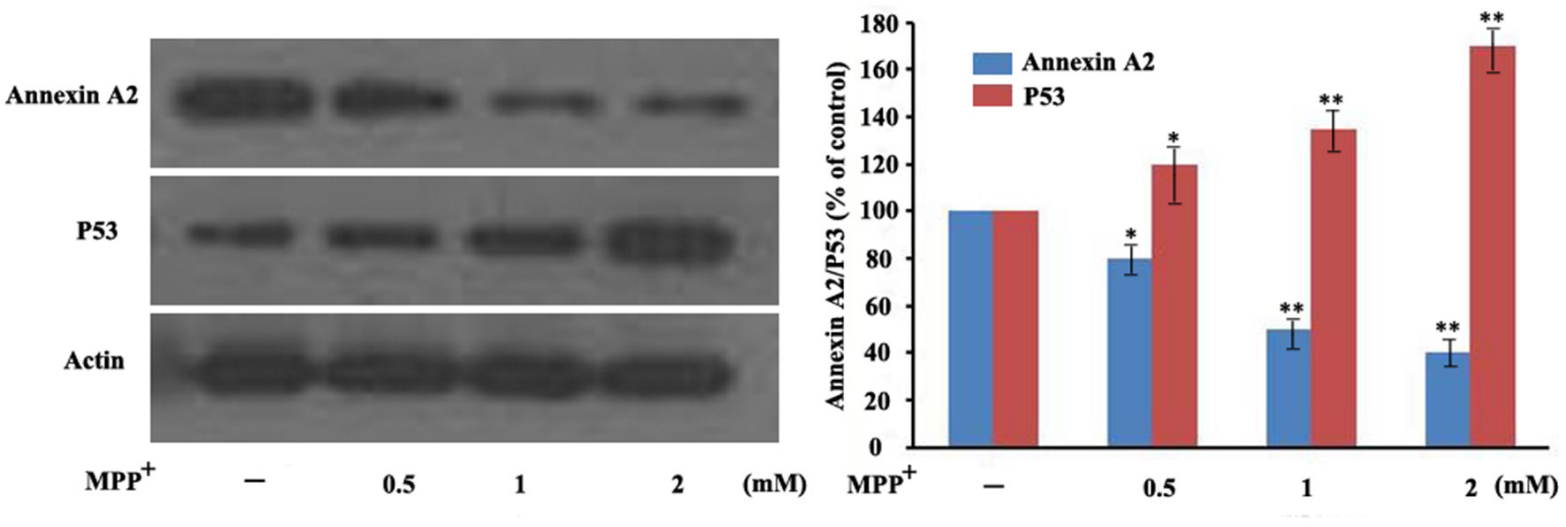
Annexin A2 expression inversely correlates with p53 levels in 1-methyl-4- phenylpyridinium (MPP^+^)-treated PC12 cells. PC12 cells were exposed to different concentrations of MPP^+^ (0.5, 1, or 2 mM) for 48 h. Annexin A2 and p53 protein levels were analyzed by western blot with β-actin as loading control. Data are presented as the mean ± SEM of three independent experiments *P < 0.05, **P < 0.01 vs. control.

Annexin A2 is a redox-sensitive protein involved in regulating mitochondrial function and ROS clearance. Given that it also downregulates p53 (Wang et al., 2012), we examined its expression in a PD cell model. Western blot analysis showed that annexin A2 levels decreased after 48 h of exposure to 0.5 mM MPP^+^, with further reductions at 1 mM and 2 mM compared to untreated controls. Concurrently, p53 expression increased inversely with annexin A2 reduction (Figure 5). These data suggest that MPP^+^-induced annexin A2 degradation may contribute to p53 upregulation in PC12 cells.

### ALA stabilizes annexin A2 to repress p53 in MPP^+^-treated PC12 cells

**Figure 6:**
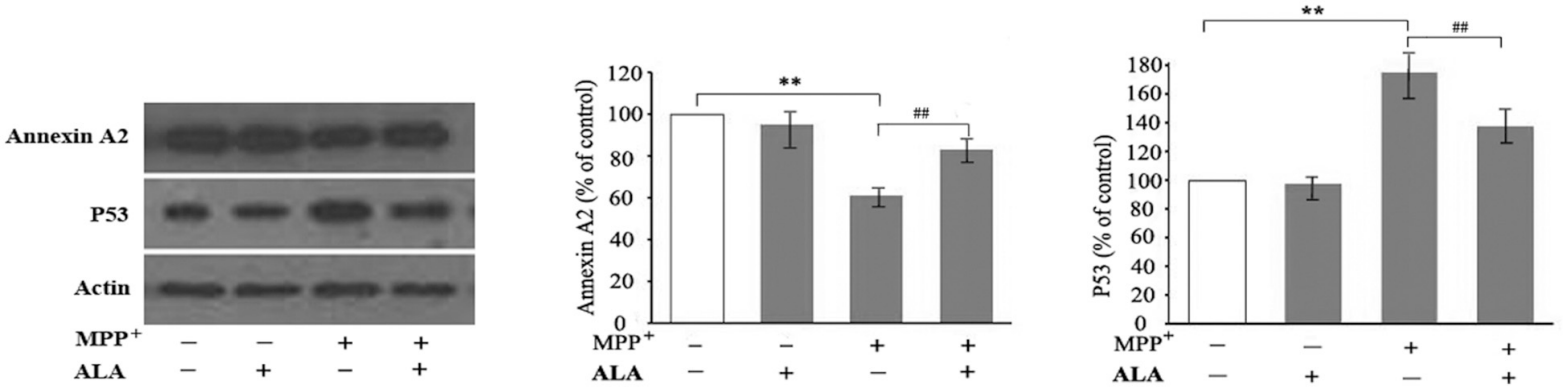
α-Lipoic acid (ALA) stabilizes annexin A2 and represses p53 in 1-methyl-4- phenylpyridinium (MPP^+^)-treated PC12 cells. PC12 cells were either untreated or treated with 2 mM MPP^+^ alone or 2 mM MPP^+^ in the presence of 0.1 μM ALA. Annexin A2 and p53 protein levels were analyzed by western blot using β-actin as loading control. Data are presented as the mean ± SEM of three independent experiments. **P < 0.01 vs. control group; ^##^*P* < 0.01 vs. MPP^+^-treated group.

To determine whether ALA regulates p53 through annexin A2, PC12 cells were pretreated with 0.1 μM ALA prior to 48 h exposure to 2 mM MPP^+^. Western blot analysis revealed that MPP^+^ significantly reduced annexin A2 protein levels (*P* < 0.01 vs. control), while ALA pretreatment substantially attenuated this reduction (*P* < 0.01 vs. MPP^+^ alone group). Concurrently, ALA co-treatment suppressed MPP^+^-induced p53 upregulation (*P* < 0.01 vs. MPP^+^ group; Figure 6). These findings demonstrate that ALA-mediated neuroprotection in dopaminergic cells involves stabilization of annexin A2, which partially represses p53 expression under neurodegenerative conditions.

## Discussion

PD is a clinically progressive movement disorder pathologically defined by degeneration of dopaminergic neurons in the SNpc (1). Current PD therapies primarily restore dopamine levels to alleviate motor symptoms transiently but fail to halt neuronal degeneration. The present investigation demonstrated that ALA significantly attenuated MPP^+^-induced cytotoxicity and apoptosis in PC12 cells. Specifically, ALA effectively reduced the degradation of annexin A2 and the overexpression of p53, both of which are important factors contributing to dopaminergic neuron degeneration and apoptosis under neurodegenerative conditions.

ALA, an essential cofactor for mitochondrial α-ketoacid dehydrogenase complexes, has been reported to enhance energy metabolism and exhibit neuroprotective properties (Li et al., 2013). In the present study, we tested ALA at concentrations ranging from 0.01 to 100 μM for its ability to protect PC12 cells against MPP^+^ toxicity. ALA at 0.1, 1, and 10 μM each conferred significant protection, with 0.1 and 1 μM showing the greatest comparable efficacy and 10 μM providing moderate protection. Based on the principle of using the minimal effective concentration to minimize potential off-target or non-specific antioxidant effects, we selected 0.1 μM for subsequent mechanistic studies. The reduced protection at 10 μM suggests a biphasic dose–response relationship, possibly due to pro-oxidant activity, cellular toxicity, or saturation of protective pathways.

p53 has been implicated in the degeneration of human substantia nigra dopamine neurons during PD pathogenesis and acts as a signaling hub mediating neuronal death in neurodegenerative disorders (Luo et al., 2022; Chen et al., 2025; Kamath et al., 2022). Substantial evidence indicates markedly elevated p53 levels and activity in cellular and animal PD models, as well as in postmortem PD brains, and these alterations correlate with mitochondrial dysfunction, oxidative stress, and neurodegeneration (Mogi et al., 2007). As a multifunctional regulator, p53 acts through both transcriptional activation of target genes and transcription-independent mechanisms (Liu et al., 2020; Vaseva et al., 2012; Talebi et al., 2021; Beckerman and Prives, 2010). Once activated, p53 translocation to mitochondria drives profound mitochondrial perturbations, including mPTP opening, Ca^2+^ overload, ROS overproduction, and impaired mitophagy, culminating in cell death (Luo et al., 2022). Mitochondrial p53 represses the antioxidant enzyme MnSOD, thereby diminishing its anti-oxidative stress activity and leading to ROS accumulation and oxidative damage (Zhao et al., 2005; Pani and Galeotti, 2011). Additionally, p53 amplifies α-synuclein-mediated neurotoxicity by enhancing its transcription while inhibiting its degradation, further aggravating mitochondrial defects and ROS generation (Duplan et al., 2016; Chen et al., 2020). Recent studies have confirmed that p53 is involved in the degeneration and apoptosis of dopaminergic neurons mediated by the TNF-α-NF-κB signaling pathway in PD, and inhibiting this axis can prevent dopamine neuron death and mitigate PD disease development (Kim et al., 2024). Consistent with these findings, our data demonstrated a dose-dependent increase in p53 expression with increasing concentrations of MPP^+^ in PC12 cells, further reinforcing its role in dopaminergic degeneration. We also found that ALA pretreatment effectively inhibited p53 overexpression and overactivation, although the underlying mechanism remains unclear.

Annexin A2 is a multifunctional protein widely expressed in eukaryotic cells and regulates diverse cellular processes, including proliferation, redox, endocytosis, exocytosis, and survival (Liu et al., 2015). Aberrant annexin A2 levels are implicated in various diseases (Hedhli et al., 2012). Notably, annexin A2 has been reported to suppress p53 expression, and its silencing markedly increases p53 protein levels (Wu et al., 2019; Li et al., 2021). The present study revealed that increasing concentrations of MPP^+^ led to a decrease in annexin A2 protein expression levels in a dose-dependent manner, suggesting the involvement of annexin A2 in the pathogenesis of PD. Western blot analysis confirmed an inverse relationship between annexin A2 and p53 expression at different concentrations of MPP^+^, indicating that annexin A2 negatively regulates p53. This negative regulation is consistent with the known function of annexin A2 in inhibiting p53 expression and activity (Wu et al., 2019; Li et al., 2021), suggesting a mechanistic link between annexin A2 and p53 in this model. Critically, ALA pretreatment effectively prevented MPP^+^-induced annexin A2 degradation, accompanied by a significant reduction in p53 protein levels. This inverse correlation suggests that ALA exerts its neuroprotective effects, at least in part, via annexin A2-dependent repression of p53.

In conclusion, this study demonstrates that ALA effectively attenuates MPP^+^-induced neurotoxicity in a PD cell model by partially stabilizing annexin A2, thereby suppressing p53 overexpression and overactivation, and consequently reducing p53-dependent dopaminergic neuron apoptosis. Although this annexin A2–p53 interaction represents a promising therapeutic target for mitigating dopaminergic degeneration, comprehensive mechanistic elucidation requires further investigation.

## Limitations

Several limitations of this study should be acknowledged. First, the experiments were conducted using a single cell line model, which may not fully recapitulate the complexity of the *in vivo* environment. Second, although our findings suggest potential involvement of oxidative stress and mitochondrial dysfunction, we did not directly measure relevant parameters such as reactive oxygen species levels, mitochondrial membrane potential, or ATP production. Third, mechanistic validation experiments, including annexin A2 knockdown/overexpression, or p53 modulation experiments, were not performed to establish causality. Future studies using multiple cell lines, *in vivo* models, and comprehensive mechanistic investigations are warranted to confirm and extend our findings.

## Data Availability

The raw data supporting the conclusions of this article will be made available by the authors, without undue reservation.
